# Electrogenerated base-promoted cyclopropanation using alkyl 2-chloroacetates

**DOI:** 10.3762/bjoc.18.114

**Published:** 2022-08-29

**Authors:** Kouichi Matsumoto, Yuta Hayashi, Kengo Hamasaki, Mizuki Matsuse, Hiyono Suzuki, Keiji Nishiwaki, Norihito Kawashita

**Affiliations:** 1 Department of Chemistry, School of Science and Engineering, Kindai University 3-4-1 Kowakae, Higashi-osaka, Osaka 577-8502, Japan; 2 Department of Pharmaceutical Sciences, Faculty of Pharmacy, Kindai University 3-4-1 Kowakae, Higashi-osaka, Osaka 577-8502, Japan; 3 Department of Life Science, School of Science and Engineering, Kindai University, 3-4-1 Kowakae, Higashi-osaka, Osaka 577-8502, Japan

**Keywords:** alkyl 2-chloroacetates, cyclopropane derivatives, divided cell, electro-reduction

## Abstract

The electrochemical reduction conditions of the reaction of alkyl 2-chloroacetates in Bu_4_NBr/DMF using a divided cell equipped with Pt electrodes to produce the corresponding cyclopropane derivatives in moderate yields were discovered. The reaction conditions were optimized, the scope and limitations, as well as scale-up reactions were investigated. The presented method for the electrochemical production of cyclopropane derivatives is an environmentally friendly and easy to perform synthetic procedure.

## Introduction

In organic chemistry, cyclopropanes and their related compounds have been recognized as important molecules. For example, cyclopropane derivatives are found in both natural products and pharmaceutical products. The cyclopropane skeleton is also found in agrochemicals, especially pyrethroid, as an insecticide, is one important compound. Cyclopropanes also play a significant role in organic synthesis as versatile building blocks [1–5]. In general, some synthetic procedures for cyclopropane derivatives have been discovered, e.g., the Simmons–Smith reaction and the use of metal carbenoids being two of the more prominent and reliable methods [6–9].

**Scheme 1:**
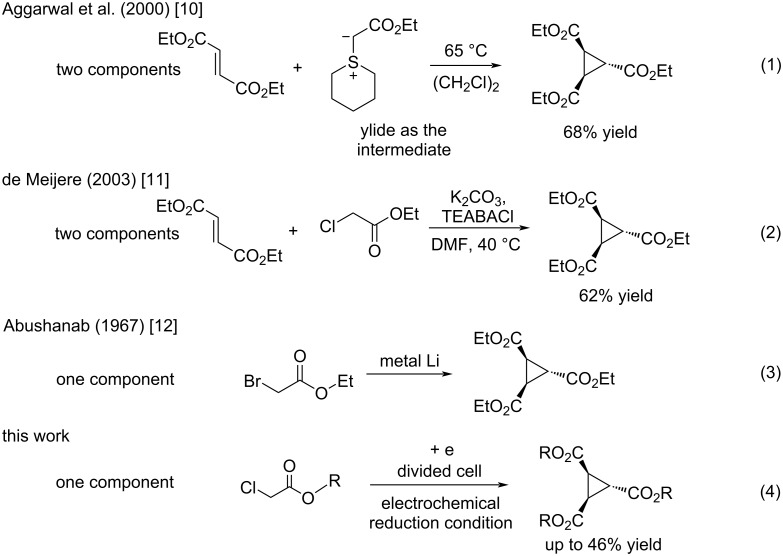
Formations of 1,2,3-trialkyl cyclopropanetricarboxylates. Previous reports (reactions 1–3) and this work (reaction 4).

Aggarwal and colleagues reported in 2000 that the reaction between a Michael acceptor such as diethyl fumarate and a sulfur-ylide, prepared from ethyl 2-diazoacetate and tetrahydro-2*H*-thiopyran in the presence of Cu(acac)_2_, yielded triethyl cyclopropane-1,2,3-tricarboxylate in 68% yield (Scheme 1, reaction 1) [10]. The same chemical yield was obtained by using a catalytic amount of tetrahydro-2*H*-thiopyran (0.2 equiv) in the process [10]. Furthermore, de Meijere and colleagues in 2003 demonstrated that the reaction of diethyl fumarate and ethyl 2-chloroacetate in DMF at 40 °C with K_2_CO_3_ and TEBACl (benzyltriethylammonium chloride) produced triethyl cyclopropane-1,2,3-tricarboxylate in 62% yield (Scheme 1, reaction 2) [11]. Both procedures are two-component coupling reactions. In contrast, a method involving a one-component reaction using alkyl 2-haloacetate has been developed by Abushanab in 1967, in which a stoichiometric amount of metal lithium was utilized to reduce ethyl 2-bromoacetate to form the corresponding 1,2,3-trisubstituted cyclopropane derivatives (Scheme 1, reaction 3) [12]. The generation of an anionic intermediate was indicated. During our study, we discovered that 1,2,3-trisubstituted cyclopropane derivatives could be formed in moderate yields through the electrochemical reduction [13–21] of alkyl 2-chloroacetates in a divided cell (Scheme 1, reaction 4). The in Abushanab’s study utilized metal lithium is one of the rarest and most expensive metals. In addition, the treatment of metal lithium is difficult and occasionally dangerous, and the reaction also produces the corresponding Li salt as waste [22–23]. In contrast, in this work, we use basic electricity to make the corresponding cyclopropane derivatives. Herein, we would like to report the details of our investigation.

## Results and Discussion

First, we investigated the reaction conditions for the electrochemical reduction to optimize the reaction outcome. The typical procedure is as follows: the electrochemical reduction was carried out in an H-type divided cell. Both electrodes were made from Pt plates. In the cathodic chamber, **1** (0.5 mmol) was dissolved in 0.3 M Bu_4_NBr in DMF (4.0 mL) and 0.3 M Bu_4_NBr in DMF (4.0 mL) was introduced to the anodic chamber. Constant current electrolysis at 12 mA until 1.0 F/mol was consumed in the cathode yielded the corresponding compound **2** in a 46% yield (Table 1, entry 1). Various parameters were varied to increase the chemical yield, as shown in Table 1. For example, the use of carbon felt as the cathode produced **2** in ≪25% yield (Table 1, entry 2). The small influence of the anodic electrode material was confirmed in the reaction using carbon felt as the anode. In this reaction compound **2** was obtained in 40% yield (Table 1, entry 3). The use of DMSO instead of DMF resulted in <22% yield of **2** (Table 1, entry 4). However, when MeOH was used **2** could not be obtained at all (Table 1, entry 5). Reactions with Bu_4_NCl, Bu_4_NI, and Bu_4_NBF_4_ instead of Bu_4_NBr produced the corresponding compound **2** in 35%, <21%, and 44% yields, respectively (Table 1, entries 6–8). The amount of the supporting electrolyte Bu_4_NBr, such as 0.8 equiv and 4.0 equiv instead of 2.4 equiv, appeared to have no influence, and **2** was produced at 40% and 43% yields, respectively (Table 1, entries 9 and 10). In terms of temperature and current (Table 1, entries 11–14), 6 mA at room temperature yielded the best result of 46% yield (Table 1, entry 14). The reaction did not take place in the absence of electricity (Table 1, entry 15). Based on the above optimizations, we chose the conditions given in entry 1 of Table 1 as the optimized parameters [24].

**Table 1:** Reaction optimization.

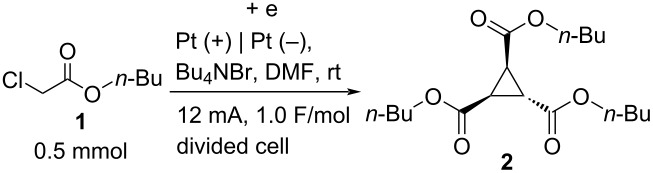		

Entry	Variation from standard conditions^a^	% Yield^b^

1	none	46
2	Pt (+) | C (–) instead of Pt (+) | Pt (–)	≪25^c^
3	C (+) | Pt (–) instead of Pt (+) | Pt (–)	40
4	DMSO as solvent^d^	<22
5	MeOH as solvent^d^	n.d.^e^
6	Bu_4_NCl instead of Bu_4_NBr^d^	35
7	Bu_4_NI instead of Bu_4_NBr^d^	<21
8	Bu_4_NBF_4_ instead of Bu_4_NBr^d^	44
9	0.8 equiv Bu_4_NBr instead of 2.4 equiv Bu_4_NBr^d^	40
10	4.0 equiv Bu_4_NBr instead of 2.4 equiv Bu_4_NBr^d^	43
11	0 °C instead of rt	45
12	60 °C instead of rt	<44
13	20 mA instead of 12 mA, 1.0 F/mol	<33
14	6 mA instead of 12 mA, 1.0 F/mol	46
15	no electric current	n.d.^c,e^

^a^Standard conditions: **1** (0.5 mmol), 0.3 M Bu_4_NBr in DMF (4.0 mL × 2), divided cell, 12 mA, rt, 1.0 F/mol of electricity against 0.5 mmol of substrate **1**. ^b^Isolated yields using preparative GPC separation of the crude materials. ^c^Observed from gas chromatography (GC) analysis. ^d^In both anodic and cathodic chambers. ^e^n.d. = no detection.

Next, we investigated the effect of electricity around 1 F/mol on the yield, as shown in Table 2, using the optimized conditions [25]. The yield of **2** was 46% in the case of 1.0 F/mol (Table 2, entry 1), as shown in entry 1 of Table 1. The chemical output of **2** was 44% in the case of 0.90 F/mol (Table 2, entry 2). However, using 1.1 F/mol resulted as well in a lower yield of **2** (<35%, Table 2, entry 3). Thus, 0.90 F/mol or 1.0 F/mol of electricity for the current reaction was found to be sufficient to obtain the product in high yield, and we choose 1.0 F/mol of electricity for the next investigations (Table 3). Finally, the electrolysis using the undivided cell shown in entry 4 of Table 2 yielded **2** in ≪5% yield, indicating that the divided cell is essential for the current reaction. In the undivided cell, the anionic species from the cathode might be consumed on the surface of the anode.

**Table 2:** Effect of electricity around 1 F/mol and type of electrochemical cell.

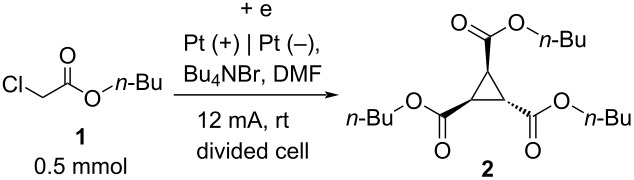		

Entry	F/mol	% Yield^a^

1	1.0	46^b^
2	0.90	44
3	1.1	<35
4^c^	1.0	≪5

^a^Isolated yields using preparative GPC separation of the crude materials. Compound **2** of entry 3 contained a small amount of impurity. ^b^This yield is from entry 1 of Table 1. ^c^An undivided cell was used instead of a divided cell.

To examine the scope and limitations, we carried out electrochemical reductions of various alkyl 2-haloacetates under the optimized conditions. Table 3 summarizes the results. The reaction of methyl 2-chloroacetate (**3**) afforded the corresponding compound **4** in 28% yield (Table 3, entry 1). The reaction of ethyl 2-chloroacetate (**5**) produced the corresponding compound **6** in a similar <23% yield (Table 3, entry 2). Ethyl 2-bromoacetate (**7**) and ethyl 2-iodoacetate (**8**), in which the leaving groups were changed from Cl to Br and I, showed similar reactivities to produce compound **6** in <24% and <26% yields, respectively (Table 3, entries 3 and 4). *n*-Propyl 2-chloroacetate (**9**), with the longer alkyl chain, and *tert*-butyl 2-chloroacetate (**11**), with the bulky alkyl group, produced **10** and **12** in <22% and <31% yields, respectively (Table 3, entries 5 and 6). The reaction of **13** with the vinyl group did not occur (Table 3, entry 7), but the reaction of compound **15** with the allyl group formed **16** in 34% yield (Table 3, entry 8). Finally, benzyl 2-chloroacetate (**17**) produced the corresponding compound **18** in 31% yield (Table 3, entry 9).

**Table 3:** Scope and limitations.

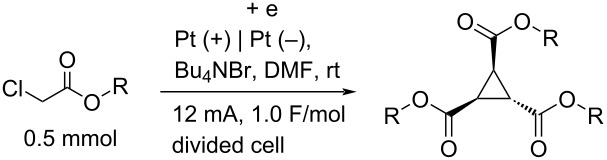					

Entry	Ester		Product		% Yield^a^

1	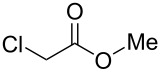	**3**	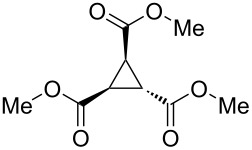	**4**	28
2	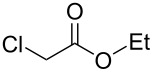	**5**	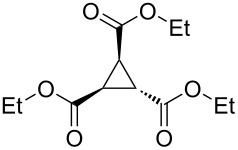	**6**	<23 (21)
3	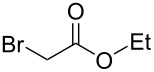	**7**		**6**	<24 (19)
4	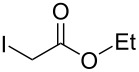	**8**		**6**	<26 (20)
5	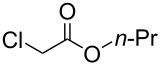	**9**	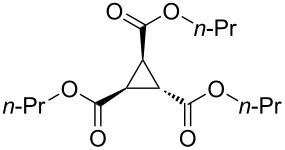	**10**	<22 (20)
6	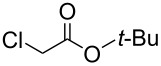	**11**	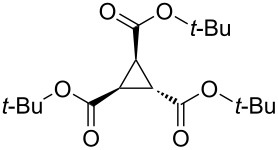	**12**	<31 (28)
7	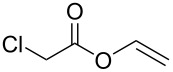	**13**	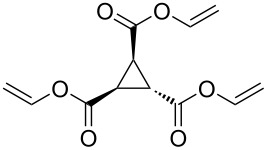	**14**	n.d.^b^
8	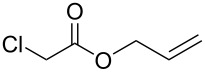	**15**	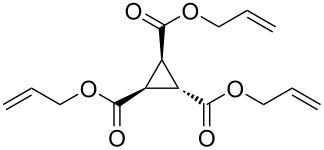	**16**	34
9	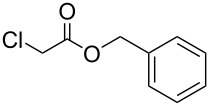	**17**	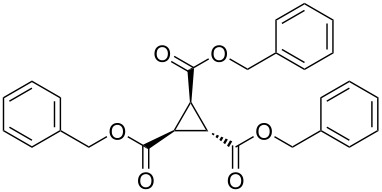	**18**	31^c^

^a^Isolated yields using preparative GPC separation of the crude materials. Compound **6** in entries 3 and 4 contained an impurity of non-negligible amount, despite of repeated purification by GPC. Compound **6** in entry 2, **10** in entry 5 and **12** in entry 6 contained a small amount of impurities (see ^13^C NMR spectra of compounds **6**, **10** and **12** in Supporting Information File 1). Values in parentheses in entries 2–6 are estimated yields, calculated from the ratio of isolated compounds and impurities given in the ^1^H NMR spectra, because the impurities seem to be the corresponding trialkyl propane-1,2,3-carboxylates (vide infra). ^b^n.d. = no detection. ^c^Isolated yield after silica-gel column chromatography.

The current electrolysis reaction can be easily scaled-up with obtaining similar yields of the products. The reaction of **1** (1.2 g, 8.0 mmol) in Bu_4_NBr/DMF at room temperature with 12 mA and 1.0 F/mol yielded the corresponding compound **2** in <45% yield (Table 4, entry 1). In addition, the reaction of **3** (1.3 g, 12.0 mmol) yielded **4** in 32% yield (Table 4, entry 2).

**Table 4:** Scale-up experiments.

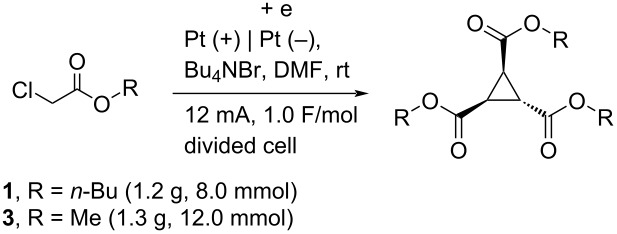				

Entry	Ester	R	Product	% Yield^a^

1	**1**	*n*-Bu	**2**	<45 (38)
2	**3**	Me	**4**	32

^a^Isolated yields using preparative GPC separation of the crude materials. Compound **2** in entry 1 contained a small amount of impurity. The value in parenthesis in entry 1 is an estimated yield, calculated from the ratio of isolated compound and impurity given in the ^1^H NMR spectrum, because the impurity seems to be tri-*n*-butyl propane-1,2,3-carboxylate (vide infra).

**Scheme 2:**
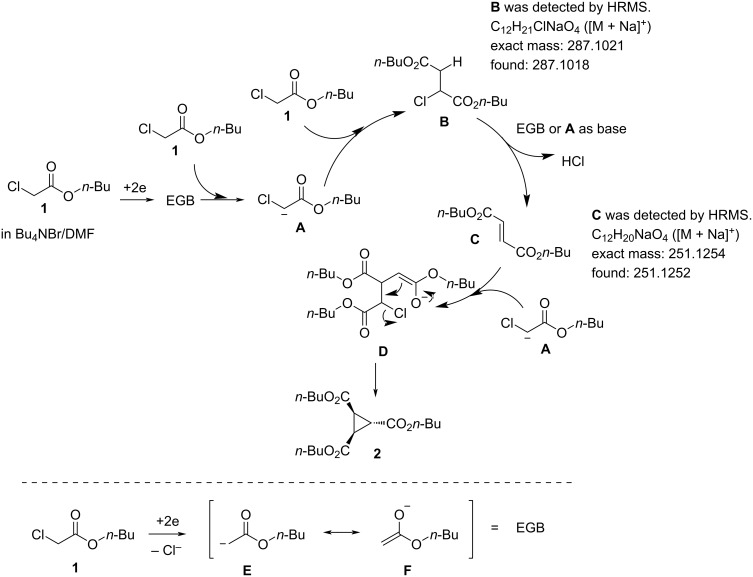
Plausible reaction mechanism. EGB = electrogenerated base.

Scheme 2 depicts a plausible reaction mechanism. We assume that the current reaction follows a similar mechanism as described in Abushanab’s report [12]. In addition, the current reaction indicated the generation of an EGB (electrogenerated base) [26–29]. The electrochemical reduction conditions of the solution containing **1** may generate an EGB, which reacts with **1** to produce anionic **A**. At the stage of the generation of the EGB, the reduction of **1** may generate an enolate ion such as **E** or **F**, which might serve as EGB, although other sources of EGBs cannot be denied [30–31]. Intermediate **A** may combine with **1** to produce **B**, which may react with the EGB or another molecule **A** to produce **C**, releasing HCl. In Abushanab’s report [12], **C** can be coupled with **A** in a similar manner to yield **D** [12]. Finally, intramolecular cyclization of **D** may yield **2**. In order to obtain a deeper insight in the reaction, we made an analysis of the crude material, which was prepared by passing 0.5 F/mol using the standard conditions and the usual work up procedure. Compounds **B** and **C** were confirmed by HRMS analysis shown in Scheme 2, which supported the current mechanism.

In addition, one of the impurities seems to be the trialkyl propane-1,2,3-carboxylates [32], because the HRMS analyses of the isolated compounds, such as **6** (Table 3, entry 2), **10** (Table 3, entry 5), and **12** (Table 3, entry 6), which were not of high purity, showed the existence of the corresponding trialkyl propane-1,2,3-carboxylates, together with the signal of the desired products **6**, **10**, and **12**. The formation of trialkyl propane-1,2,3-carboxylate might be through the Michael addition of the electrogenerated base (**E** in Scheme 2) to **C** or the electrochemical reduction of **2**.

## Conclusion

A new electrochemical transformation of alkyl 2-chloroacetates to cyclopropane derivatives has been developed. The reaction has been optimized, the scope and limitations have been investigated. Scale-up reactions were performed and satisfactory yields obtained. The generation of an EGB of the enolate ion from alkyl 2-chloroacetates is indicated. The current method is one of the most environmentally benign and accessible methods for the preparation of 1,2,3-trisubstituted cyclopropane derivatives, notwithstanding the low reaction yields. In our laboratory, further synthetic investigations are in progress.

## Supporting Information

File 1 Experimental details, characterization data of new compounds and copies of ^1^H NMR and ^13^C NMR spectra.
