# Supplementary material for: Electrogenerated base-promoted cyclopropanation using alkyl 2-chloroacetates
Source: Beilstein J Org Chem. 2022 Aug 29;18:1116–22. doi: 10.3762/bjoc.18.114 (PMC9443391; doi:10.3762/bjoc.18.114)
Supplement: File 1 — Experimental details, characterization data of new compounds and copies of 1H NMR and 13C NMR spectra. [file Beilstein_J_Org_Chem-18-1116-s001.pdf]

BEILSTEIN JOURNAL OF ORGANIC CHEMISTRY

Supporting Information

for

Electrogenerated base-promoted cyclopropanation using alkyl 2-chloroacetates

Kouichi Matsumoto, Yuta Hayashi, Kengo Hamasaki, Mizuki Matsuse, Hiyono Suzuki, Keiji Nishiwaki and Norihito Kawashita

Beilstein J. Org. Chem. **2022,** *18,* 1116–1122. doi:10.3762/bjoc.18.114

Experimental details, characterization data of new compounds and copies of ^{1}H NMR and ^{13}C NMR spectra

License and Terms: This is a supporting information file under the terms of the Creative Commons Attribution License (https://creativecommons.org/licenses/by/4.0). Please note that the reuse, redistribution and reproduction in particular requires that the author(s) and source are credited and that individual graphics may be subject to special legal provisions.
The license is subject to the *Beilstein Journal of Organic Chemistry* terms and conditions: (https://www.beilstein-journals.org/bjoc/terms)

1. General remarks

^{1}H NMR and ^{13}C NMR spectra were carried out by using a Varian MERCURY 300 (^{1}H NMR 300 MHz, ^{13}C NMR 75 MHz) and a JEOL JNM-ECS 400 (^{1}H NMR 400 MHz, ^{13}C NMR 100 MHz) spectrometer. Unless otherwise noted, $CDCl_3$ was used as the solvent. In ^{1}H NMR, residual solvent ($CHCl_3$, 7.26 ppm) or tetramethylsilane (0.00 ppm) was used as internal standard. In ^{13}C NMR of $CDCl_3$, the chemical shift is referenced to the signal at 77.0 ppm. High-resolution mass spectra (HRMS) were measured using a Thermo Fisher Scientific Exactive Plus spectrometer. Merck pre-coated silica gel F_{254} plates (thickness 0.25 mm) were used for the TLC analysis. A silica gel column (Kanto Chem. Co., Silica Gel N, spherical, neutral, 40–100 μm) was used for the flash chromatography using an air pump. An LC-9201, LC-9110 NEXT, or LC-9210 NEXT apparatus, equipped with JAIGEL-1H and JAIGEL-2H, was used for the preparative GPC separation with recycle mode, and $CHCl_3$ was used as an eluent. Gas chromatography was performed by using a Shimadzu GC-2014 apparatus, equipped with a capillary column and FID. Electrochemical reactions were carried out using a power supply such as a KIKUSUI PMC350-0.2A and PMX350-0.2A. All reactions were conducted under an N_2 atmosphere.

2. Materials

Dry solvents DMF (*N*,*N*-dimethylformamide), DMSO (dimethyl sulfoxide) and MeOH (methanol), and Bu_4NBF_4 (tetrabutylammonium tetrafluoroborate), Bu_4NCl (tetrabutylammonium chloride), Bu_4NI (tetrabutylammonium iodide) and Bu_4NBr (tetrabutylammonium bromide) were obtained from commercial suppliers. Bu_4NBF_4 was dried at 50 °C under reduced pressure (1 mmHg) overnight before use. The esters such as *n*-butyl 2-chloroacetate (**1**), methyl 2-chloroacetate (**3**), ethyl 2-chloroacetate (**5**), ethyl 2-bromoacetate (**7**), ethyl 2-iodoacetate (**8**), *tert*-butyl 2-chloroacetate (**11**), vinyl 2-chloroacetate (**13**), allyl 2-chloroacetate (**15**), and benzyl 2-chloroacetate (**17**) were obtained from commercial suppliers, and used without the further purification.

3. Synthesis of *n*-propyl 2-chloroacetate (9)

A three-necked round flask was dried by heating gun under reduced pressure, and filled afterwards with N_2.

To the flask, chloroacetic acid (9.45 g, 100 mmol), *n*-propanol (100 mL), and 98% sulfuric acid (2.0 mL) were added. The solution was refluxed for 24 h. After the reaction, the solution was evaporated under reduced pressure to remove the solvent. Then, the residue was poured into H_2O (250 mL), and

the mixture was neutralized by the addition of aq. 10% $NaHCO_3$. The mixture was extracted with Et_2O (20 mL × 3), and the combined organic phase was washed with H_2O (30 mL × 2). The combined organic phase was dried over $MgSO_4$, filtered and concentrated under reduced pressure. The crude material was purified by distillation under atmospheric pressure to give *n*-propyl 2-chloroacetate (**9**, 2.80 g, 20.5 mmol, 21% yield), which was identified by comparison of the reported spectroscopic data [1].

4. General procedure for the formation of cyclopropane derivatives (Table 1, entry 1).

Cathodic reduction was carried out in an H-type divided cell (4G glass filter) equipped with two platinum plates in anode and cathode (1 cm × 2 cm). After drying and replacement by N_2, 0.3 M Bu_4NBr/DMF (4.0 mL × 2) was added to anode and cathode. In the cathodic chamber, **1** (75.6 mg, 0.502 mmol) was added and stirred. The reduction was carried out by using 12 mA until 1.0 F/mol was consumed. After the electrolysis, the solutions of the cathode and anode were collected and poured into 10% $Na_2S_2O_3$ (20 mL) and hexane/AcOEt (4:1) (10 mL). Additional hexane/AcOEt (4:1) (10 mL) was used to collect the organic materials from the H-type divided cell. To the mixture, hexane/AcOEt (4:1) (20 mL) was added and separated. The aqueous phase was extracted with hexane/AcOEt (4:1) (20 mL × 2). The combined organic phase was washed by brine (10 mL), dried over Na_2SO_4, filtered and concentrated. A short column was also carried out with hexane/AcOEt (3:1). The solution was concentrated to give the crude material, which was purified by using preparative GPC separation with recycle mode to give **2** (26.1 mg, 0.0762 mmol, 46% yield). ^{1}H NMR (400 MHz, $CDCl_3$) δ 0.89-0.98 (m, 9H), 1.32-1.45 (m, 6H), 1.56-1.68 (m, 6H), 2.54 (d, *J* = 5.6 Hz, 2H), 2.77 (t, *J* = 5.6 Hz, 1H), 4.05-4.16 (m, 6H) ppm; ^{13}C NMR (100 MHz, $CDCl_3$) δ 13.7, 19.01, 19.04, 25.6, 28.5, 30.5, 65.4, 65.5, 167.7, 170.2 ppm; HRMS (ESI) calculated for $C_{18}H_{31}O_6$ ($[M+H]^+$): 343.2115, found 343.2103.

***trans*-Trimethyl cyclopropane-1,2,3-tricarboxylate (4).** ^{1}H NMR (400 MHz, $CDCl_3$) δ 2.56 (d, *J* = 5.6 Hz, 2H), 2.80 (t, *J* = 5.6 Hz, 1H), 3.72 (s, 6H), 3.74 (s, 3H) ppm; ^{13}C NMR (100 MHz, $CDCl_3$) δ 25.6, 28.2, 52.6, 168.0, 170.4 ppm; HRMS (ESI) calculated for $C_9H_{12}O_6Na$ ($[M+Na]^+$): 239.0526, found 239.0521.

***trans*-Triethyl cyclopropane-1,2,3-tricarboxylate (6).** ^{1}H NMR (400 MHz, $CDCl_3$) δ 1.23-1.32 (m, 9H), 2.54 (d, *J* = 5.6 Hz, 2H), 2.77 (t, *J* = 5.6 Hz, 1H), 4.11-4.23 (m, 6H) ppm; ^{13}C NMR (100 MHz,

$CDCl_3$) δ 14.1, 25.6, 28.5, 61.5, 61.6, 167.6, 170.1 ppm; HRMS (ESI) calculated for $C_{12}H_{18}O_6Na$ ($[M+Na]^+$): 281.0996, found 281.0990.

6 was known compound, and **6** was identified by comparison of the reported spectroscopic data [2].

***trans*-Tripropyl cyclopropane-1,2,3-tricarboxylate (10).** ^{1}H NMR (400 MHz, $CDCl_3$) δ 0.90-1.0 (m, 9H), 1.58-1.74 (m, 6H), 2.55 (d, *J* = 5.6 Hz, 2H), 2.78 (t, *J* = 5.6 Hz, 1H), 4.01-4.12 (m, 6H) ppm; ^{13}C NMR (100 MHz, $CDCl_3$) δ 10.28, 10.31, 21.8, 21.9, 25.6, 28.5, 67.1, 167.7, 170.2 ppm; HRMS (ESI) calculated for $C_{15}H_{24}O_6Na$ ($[M+Na]^+$): 323.1465, found 323.1456.

***trans*-Tri-*tert*-butyl cyclopropane-1,2,3-tricarboxylate (12).** ^{1}H NMR (400 MHz, $CDCl_3$) δ 1.450 (s, 9H), 1.455 (s, 18H), 2.36 (d, *J* = 5.6 Hz, 2H), 2.58 (t, *J* = 5.6 Hz, 1H) ppm; ^{13}C NMR (100 MHz, $CDCl_3$) δ 26.3, 27.97, 28.00, 29.6, 81.7, 166.8, 169.6 ppm; HRMS (ESI) calculated for $C_{18}H_{30}O_6Na$ ($[M+Na]^+$): 365.1935, found 365.1931.

***trans*-Triallyl cyclopropane-1,2,3-tricarboxylate (16).** ^{1}H NMR (400 MHz, $CDCl_3$) δ 2.61 (d, *J* = 5.6 Hz, 2H), 2.85 (t, *J* = 5.6 Hz, 1H), 4.58-4.64 (m, 6H), 5.22-5.39 (m, 6H), 5.84-5.98 (m, 3H) ppm; ^{13}C NMR (100 MHz, $CDCl_3$) δ 25.7, 28.5, 66.2, 118.9, 119.1, 131.4, 131.5, 167.1, 169.6 ppm; HRMS (ESI) calculated for $C_{15}H_{18}O_6Na$ ($[M+Na]^+$): 317.0996, found 317.0977.

***trans*-Tribenzyl cyclopropane-1,2,3-tricarboxylate (18).** ^{1}H NMR (400 MHz, $CDCl_3$) δ 2.63 (d, *J* = 5.6 Hz, 2H), 2.89 (t, *J* = 5.6 Hz, 1H), 5.04 (s, 4H), 5.12 (s, 2H), 7.27-7.40 (m, 15H) ppm; ^{13}C NMR (100 MHz, $CDCl_3$) δ 25.7, 28.6, 67.3, 67.4, 128.4, 128.50, 128.54, 128.6, 135.0, 135.2, 167.2, 169.7 ppm; HRMS (ESI) calculated for $C_{27}H_{24}O_6Na$ ($[M+Na]^+$): 467.1465, found 467.1458.

5. Gram-scale synthesis of 2 from 1 (Table 4, entry 1).

Cathodic reduction was carried out in an H-type divided cell (4G glass filter) equipped with two platinum plates as anode and cathode (2 cm × 2 cm). After drying and replacement with N_2, 0.3 M Bu_4NBr/DMF (30 mL × 2) was added to anode and cathode. In the cathodic chamber, **1** (1.205 g, 8.0 mmol) was added and stirred. The reduction was carried out by using 12 mA until 1.0 F/mol was consumed. After the electrolysis, the solutions of the cathode and the anode were collected and poured into 10% $Na_2S_2O_3$ (150 mL) and hexane/AcOEt (4:1) (20 mL). Additional hexane/AcOEt (4:1) (20 mL) was used to collect the organic materials from the H-type divided cell. To the mixture,

hexane/AcOEt (4:1) (50 mL) was added and separated. The aqueous phase was extracted with hexane/AcOEt (4:1) (50 mL × 2). The combined organic phase was washed with brine (30 mL), and dried over Na_2SO_4, filtered and concentrated. A short column was also carried out with hexane/AcOEt (3:1). The solution was concentrated to give the crude material, which was purified by using preparative GPC separation with recycle mode to give **2** (409.8 mg, 1.20 mmol, 45% yield), in which the connected JAIGEL-1H-40 and JAIGEL-2H-40 were used.

References

[1] Oliveira, R. F.; Souza, H. D. S.; Alves, F. S.; Sousa, A. P.; Lima, P. S. V.; Huang, M.-F. N.; Cordeiro, L. V.; Neto, H. D.; Lima, E. O.; Trindade, E. O.; Barbosa-Filho. J. M.; Athayde-Filho, P. F. *J. Braz. Chem. Soc.* **2020**, *31*, 953.

[2] Chen, Y.; Ruppel, J. V.; Zhang, X. P. *J. Am. Chem. Soc.* **2007**, *129*, 12074.

trans-Tributyl cyclopropane-1,2,3-tricarboxylate (**2**)

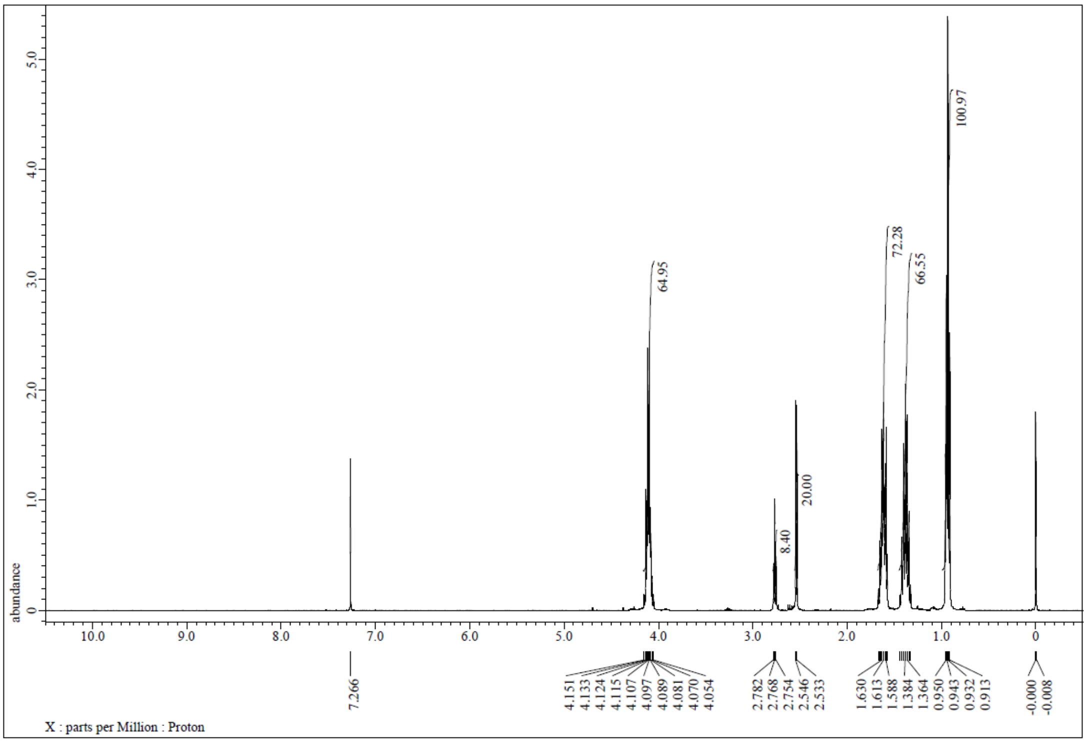

^{1}H NMR of **2**

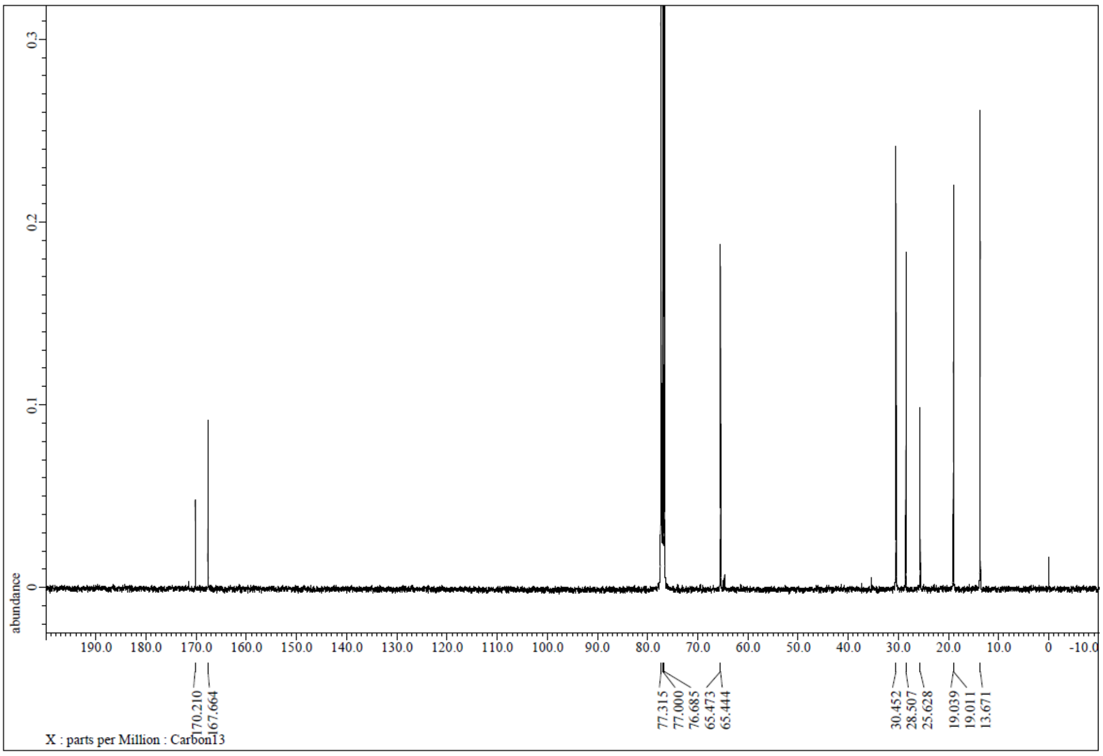

^{13}C NMR of **2**

trans-Trimethyl cyclopropane-1,2,3-tricarboxylate (**4**)

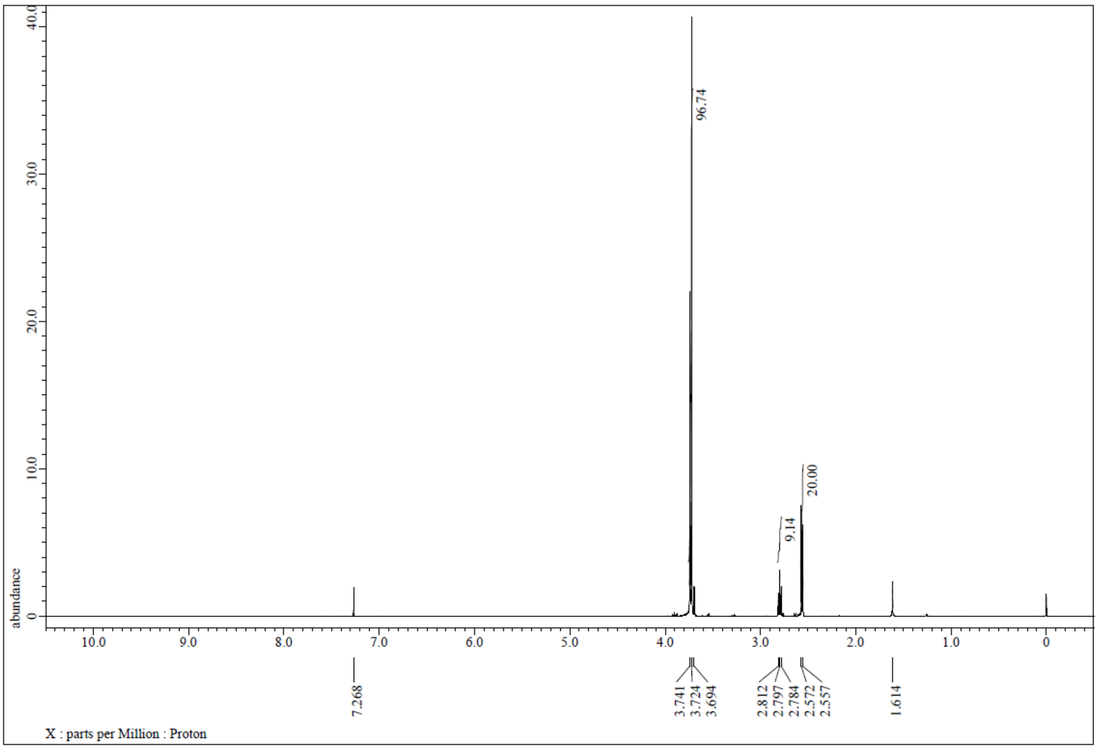

^{1}H NMR of **4**

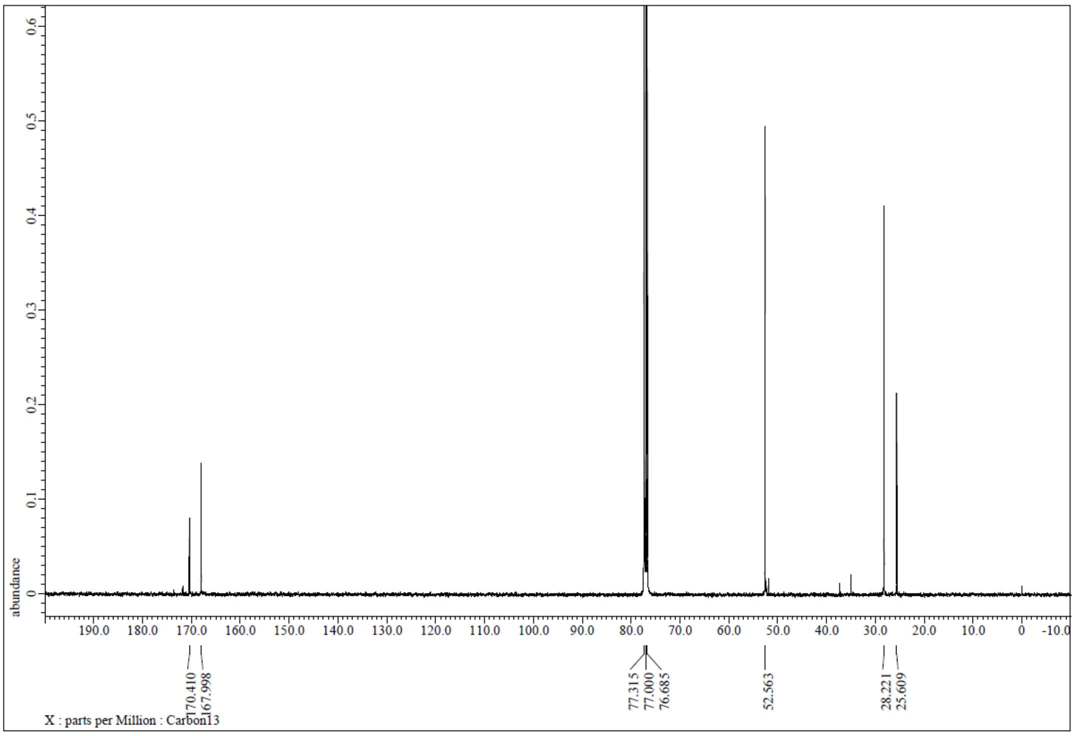

^{13}C NMR of **4**

trans-Triethyl cyclopropane-1,2,3-tricarboxylate (**6**)

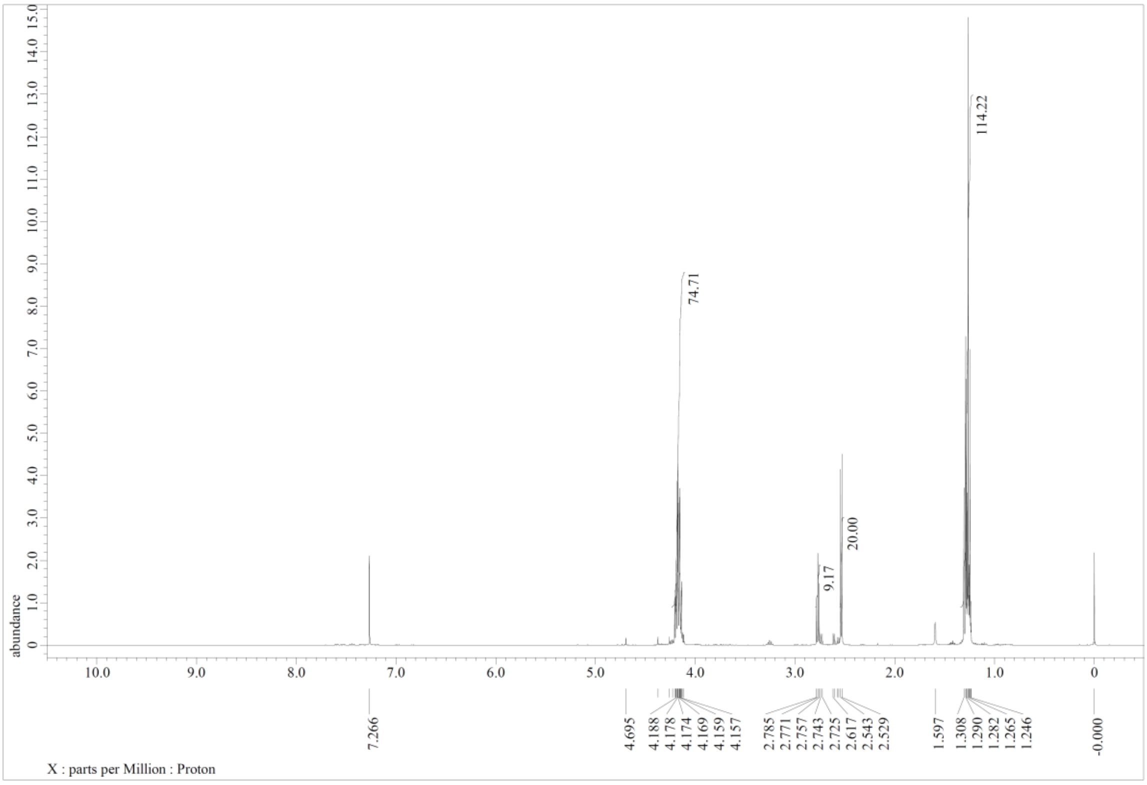

^{1}H NMR of **6** from entry 2 in Table 3

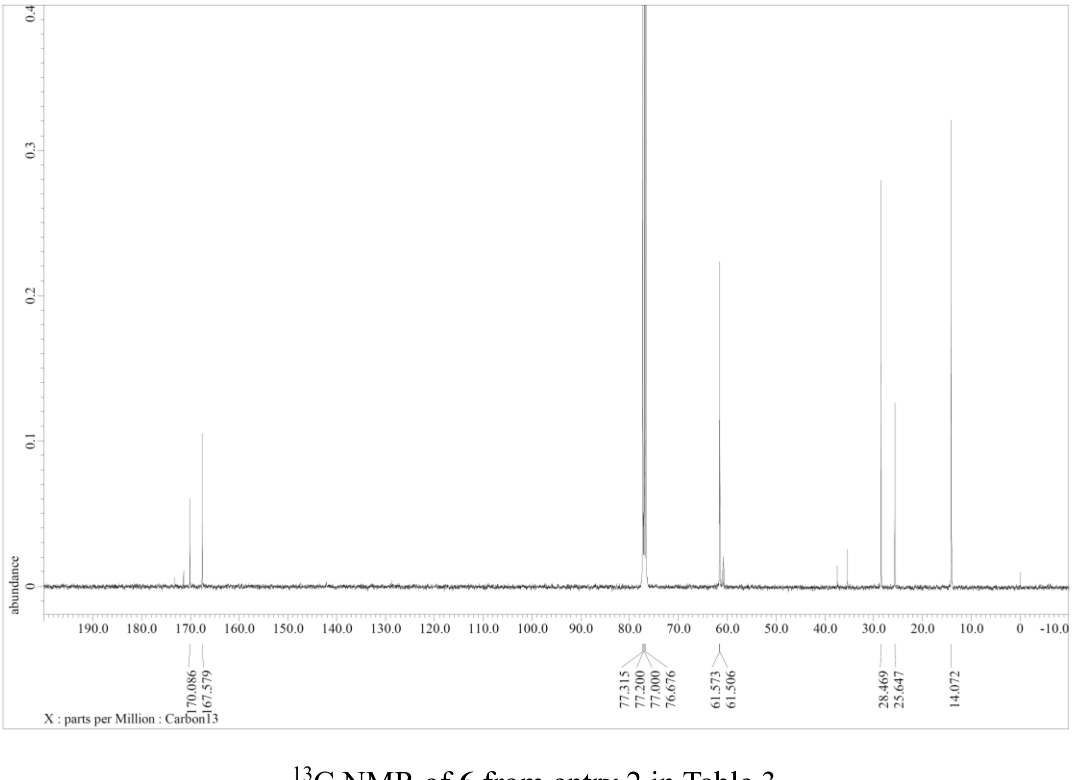

^{13}C NMR of **6** from entry 2 in Table 3

trans-Tripropyl cyclopropane-1,2,3-tricarboxylate (**10**)

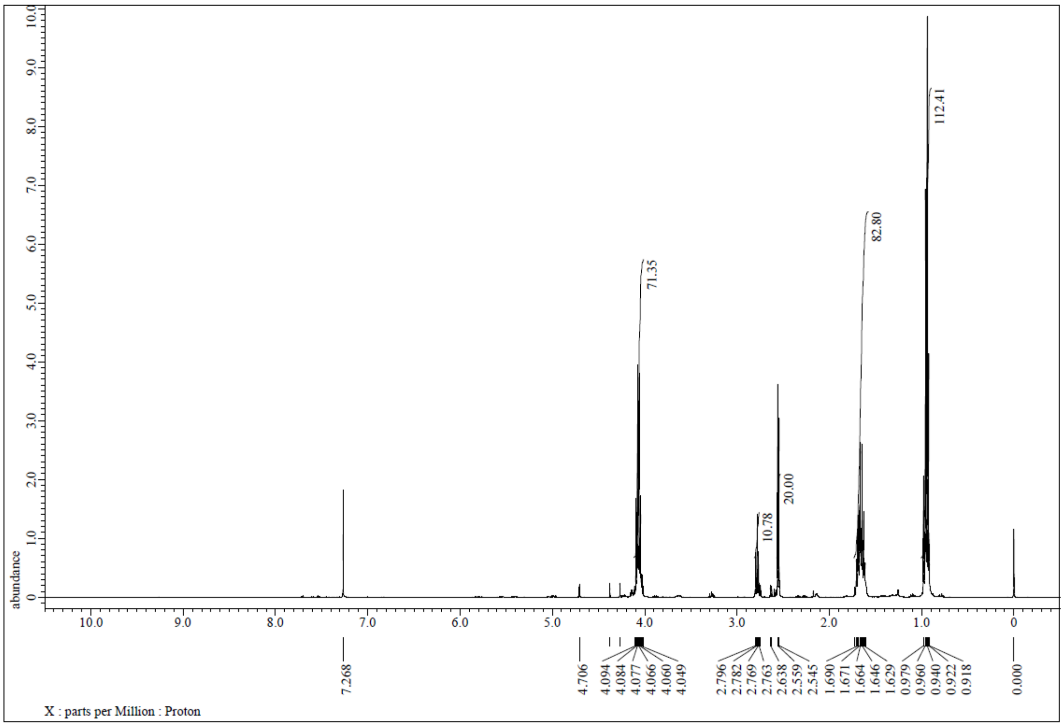

^{1}H NMR of **10**

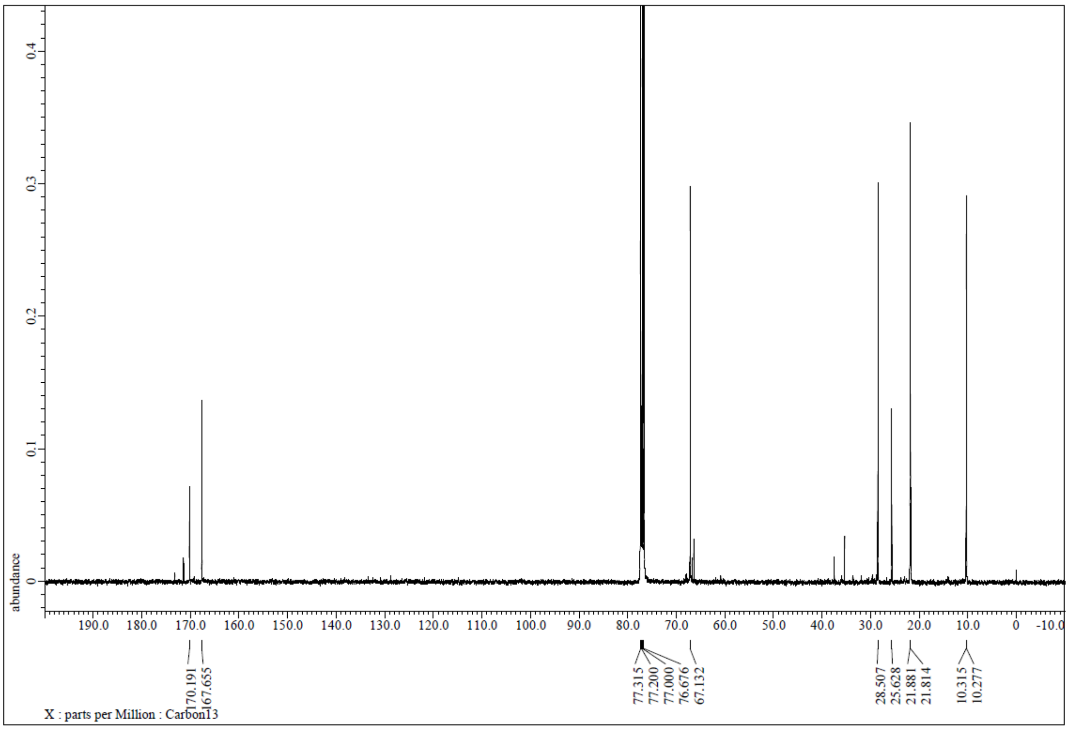

^{13}C NMR of **10**

trans-Tri-*tert*-butyl cyclopropane-1,2,3-tricarboxylate (**12**)

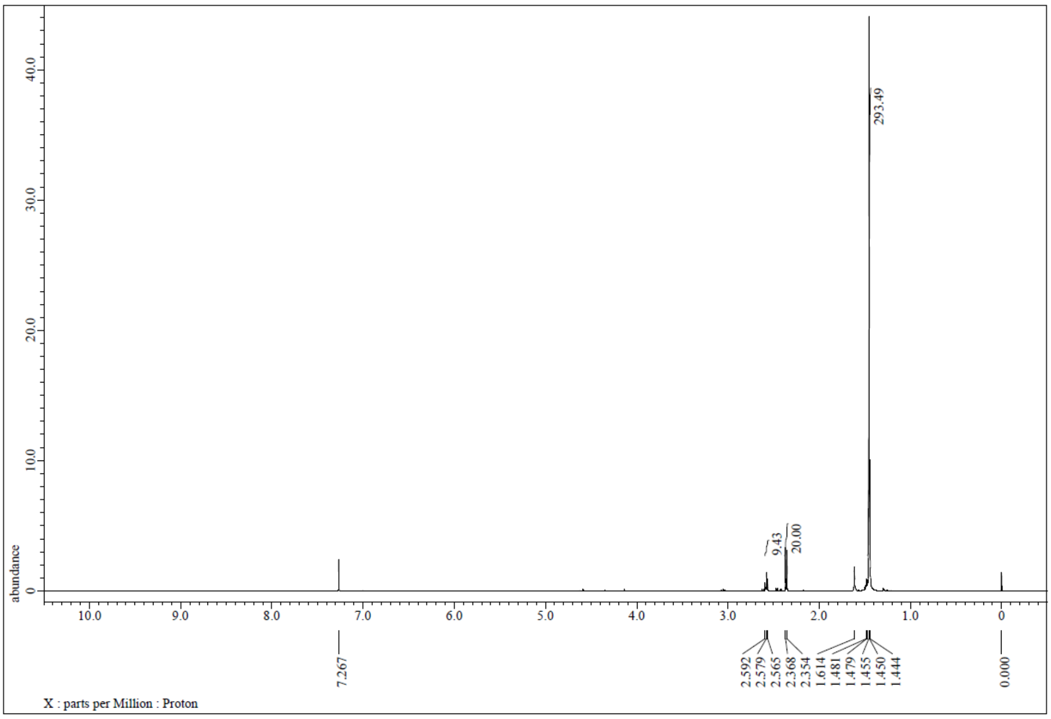

^{1}H NMR of **12**

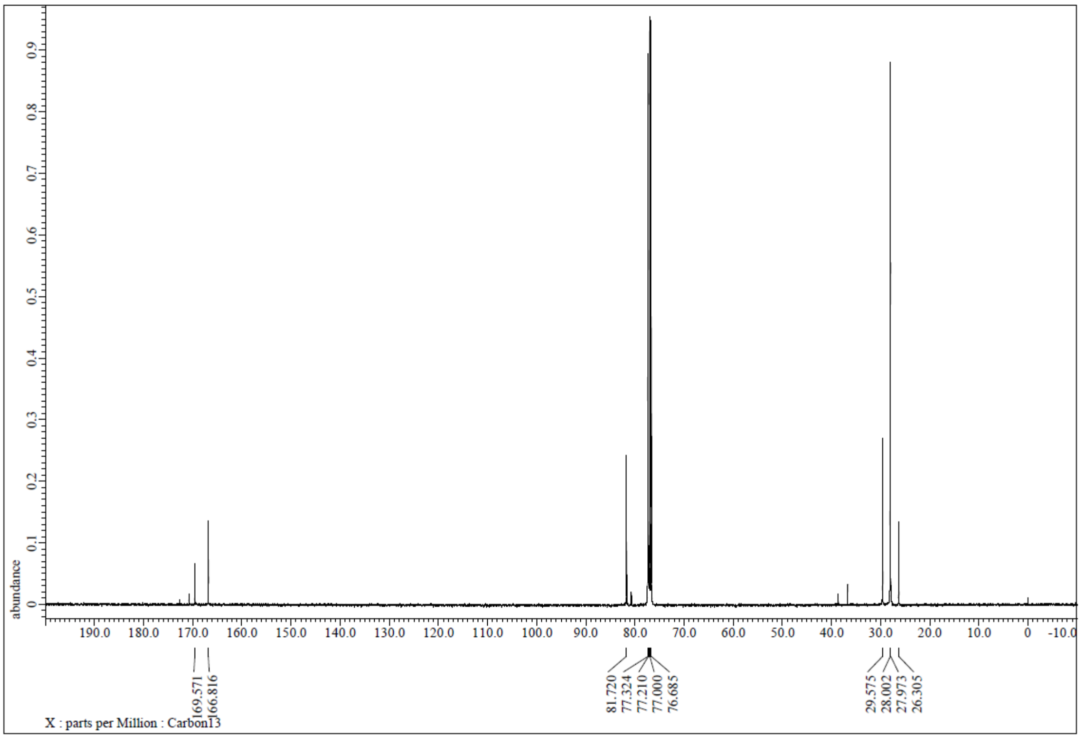

^{13}C NMR of **12**

trans-Triallyl cyclopropane-1,2,3-tricarboxylate (**16**)

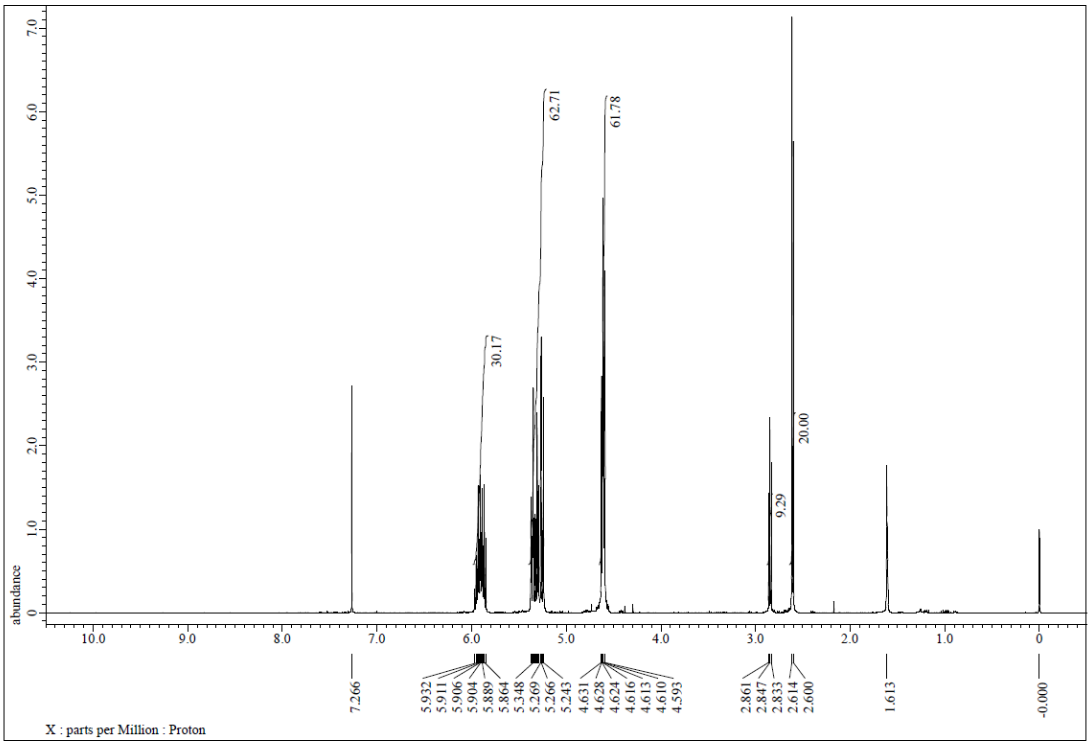

^{1}H NMR of **16**

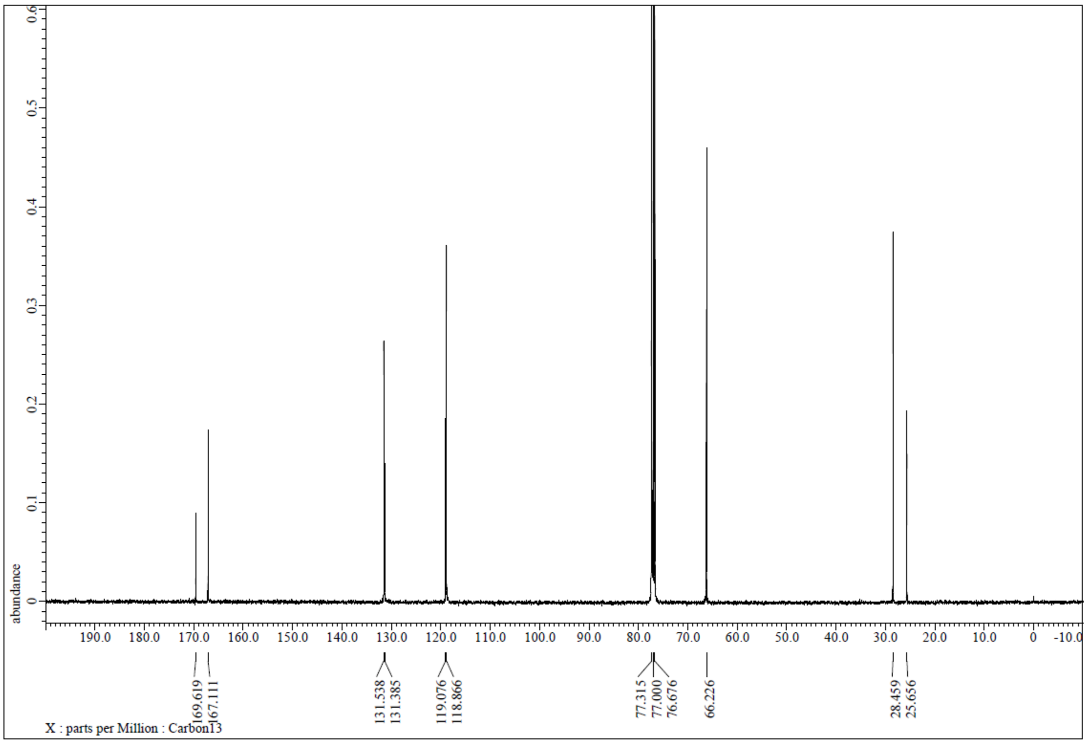

^{13}C NMR of **16**

trans-Tribenzyl cyclopropane-1,2,3-tricarboxylate (**18**)

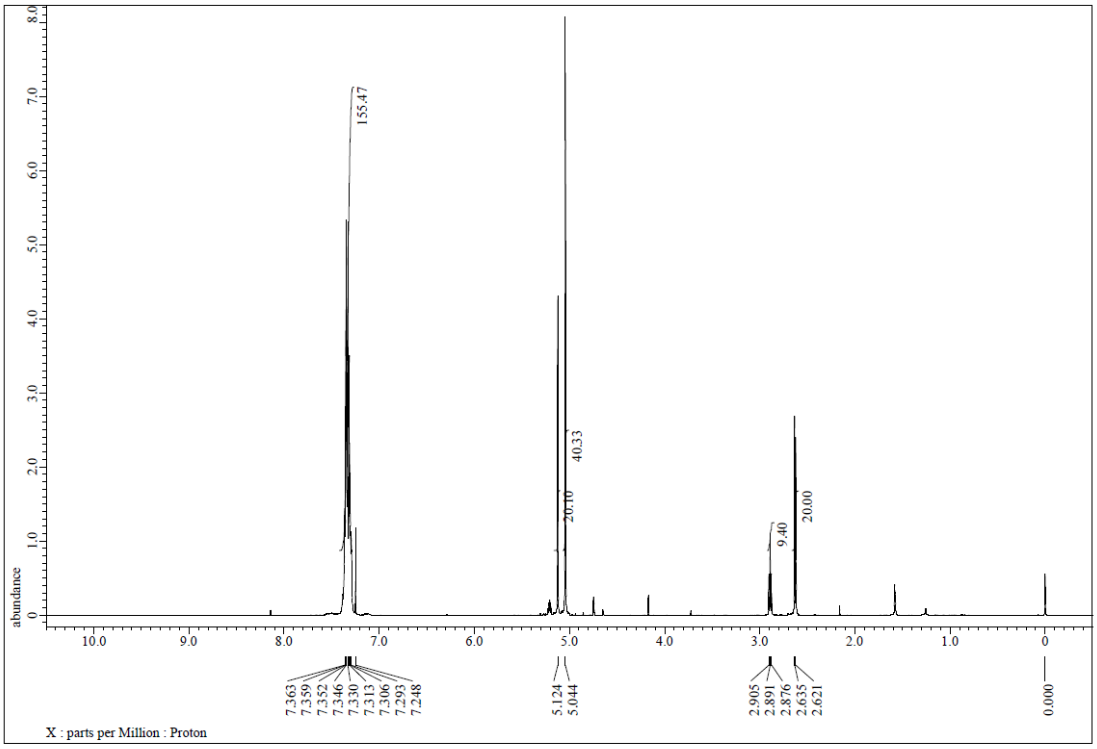

^{1}H NMR of **18**

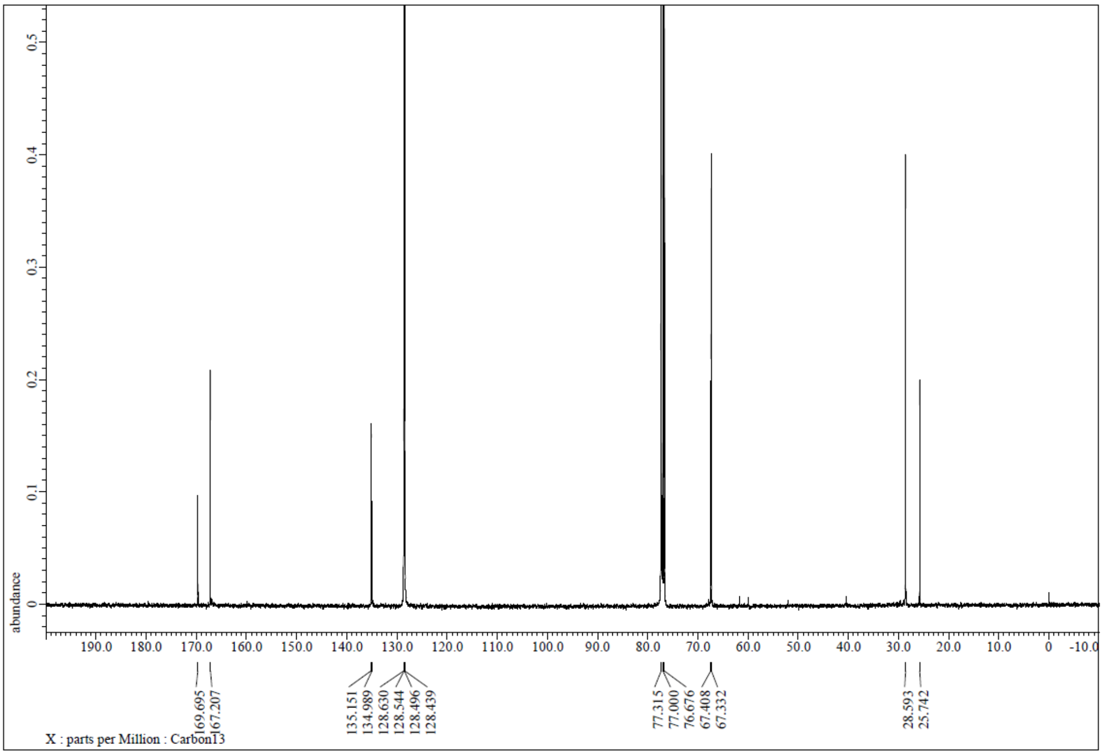

^{13}C NMR of **18**
